# Nuclear Imaging in Infective Endocarditis

**DOI:** 10.3390/ph15010014

**Published:** 2021-12-22

**Authors:** Nidaa Mikail, Fabien Hyafil

**Affiliations:** 1 Department of Nuclear Medicine, Beaujon University Hospital, Assistance Publique-Hôpitaux de Paris, 100 Boulevard du Général Leclerc, 92110 Clichy, France; nidaa.mikail@usz.ch; 2 Department of Nuclear Medicine, University Hospital Zurich, Rämistrasse 100, CH-8006 Zurich, Switzerland; 3 Center for Molecular Cardiology, University of Zurich, Wagistrasse 12, CH-8952 Schlieren, Switzerland; 4 Department of Nuclear Medicine, Georges-Pompidou European Hospital, DMU IMAGINA, Assistance Publique-Hôpitaux de Paris, University of Paris, 20 Rue Leblanc, 75015 Paris, France

**Keywords:** infective endocarditis, native valve endocarditis, prosthetic valve endocarditis, cardiac implanted electronic device, left ventricular assistance device, vascular graft infection, nuclear medicine, scintigraphy, ^18^F-FDG, positron emission tomography, white blood cell scintigraphy

## Abstract

Infective endocarditis (IE) is a life-threatening disease with stable prevalence despite prophylactic, diagnostic, and therapeutic advances. In parallel to the growing number of cardiac devices implanted, the number of patients developing IE on prosthetic valves and cardiac implanted electronic device (CIED) is increasing at a rapid pace. The diagnosis of IE is particularly challenging, and currently relies on the Duke-Li modified classification, which include clinical, microbiological, and imaging criteria. While echocardiography remains the first line imaging technique, especially in native valve endocarditis, the incremental value of two nuclear imaging techniques, ^18^F-fluorodeoxyglucose positron emission tomography with computed tomography (^18^F-FDG-PET/CT) and white blood cells single photon emission tomography with computed tomography (WBC-SPECT), has emerged for the management of prosthetic valve and CIED IE. In this review, we will summarize the procedures for image acquisition, discuss the role of ^18^F-FDG-PET/CT and WBC-SPECT imaging in different clinical situations of IE, and review the respective diagnostic performance of these nuclear imaging techniques and their integration into the diagnostic algorithm for patients with a suspicion of IE.

## 1. Introduction

Despite significant diagnostic and therapeutic progresses, infective endocarditis (IE) remains associated with high morbidity and mortality [1,2]. IE affects 3–10/100,000/year in developed countries [3], and its incidence is growing in the United States [4]. IE-related mortality reaches 20% at 30 days [5], increasing to up to 40–50% at late follow-up [6,7]. The number of implanted cardiac devices is increasing at a rapid pace, in particular in elderly patients with multiple comorbidities. This population has a high prevalence of sepsis related to secondary infection of the implanted material [1,3,7]. The mortality of IE is related to local complications, such as valve degradation and periannular abscesses, and to distant embolization, which may be fatal, in particular in case of septic embols in the brain [3]. IE treatment may require urgent cardiac surgery, which is associated with a high risk of mortality in this context, even if performed at an early stage of the disease [8,9]. The prognosis remains particularly poor in patients with IE-related stroke, despite adequate reperfusion therapy [10,11].

The diagnosis of IE is challenging. Establishing an IE diagnosis is currently based on the Duke-Li criteria (Table 1), which combine clinical, biological/microbiological, and imaging parameters [12]. Based on these criteria, the diagnosis of IE is classified as *definite*, *possible*, or *rejected* (Table 2). Given the non-specific value of most clinical and biological criteria, imaging plays a central role in IE management. While echocardiography remains the mainstay exam, in particular for native valve endocarditis (NVE), its diagnostic performance is lower in prosthetic valves endocarditis (PVE) [13], because of acoustic shadowing due to the material and the difficulty to identify perivalvular infection [14]. This also holds true for transesophageal echocardiography (TEE), which despite having higher performances than transthoracic echography (TTE), does not allow ruling out PVE with high confidence in case of negative findings [15,16]. This can delay the diagnosis and the treatment initiation, resulting in poorer clinical outcome [17]. Thus, advanced noninvasive imaging techniques are increasingly used in the management of IE, particularly in case of discordance between the clinical presentation and echocardiography, or in situations where the diagnosis is deemed *possible* based on the Duke-Li criteria [18]. Nuclear medicine imaging techniques, i.e., ^18^Fluor radiolabeled fluorodeoxyglucose positron emission tomography combined with computed tomography (^18^F-FDG-PET/CT), and white blood cell (WBC) scintigraphy provide high sensitivity (Se) for the detection of infective foci and have demonstrated their incremental value over TEE for the diagnostic of PVE (Table 3). The European guidelines for the management of IE have indeed modified the Duke-Li criteria, incorporating intracardiac findings from ^18^F-FDG-PET/CT and WBC scintigraphy as major criteria of IE [12]. Following on the modified Duke-Li criteria and the European Society of Cardiology criteria for IE, the International CIED Infection Criteria have also been developed in 2019 [19] (Table 4). Non-nuclear medicine imaging techniques, i.e., cardiac computed tomography angiography and cardiac magnetic resonance imaging also play a critical role in the diagnosis of IE. The main specificities of each technique are listed in Table 5.

In this review, we will discuss the role of ^18^F-FDG-PET/CT and WBC single photon emission computed tomography (WBC-SPECT) imaging for the different clinical presentations of IE and review their respective diagnostic performance. We will also summarize the practical approach of nuclear imaging in suspected IE, as well as the diagnostic algorithm recommended by the latest available guidelines.

## 2. Rationale for the Use of Nuclear Medicine Imaging

### 2.1. F-FDG PET

^18^F-FDG is a radioactive analog of glucose, in which a hydroxyl group has been replaced by ^18^F, a positron-emitting radionuclide [20]. Similar to glucose, ^18^F-FDG enters the cell via GLUT membrane transporters, thereby indicating cells with increased metabolic activity. However, unlike glucose, ^18^F-FDG does not undergo further glycolysis, which is blocked by ^18^F. Consequently, ^18^F-FDG accumulates in the cell—a phenomenon coined metabolic trapping. Therefore, the concentration of ^18^F-FDG reflects the actual concentration of glucose in the tissue, enabling an absolute quantification of its metabolic activity. Owing to this, the higher the metabolic activity of the tissue, the higher the accumulation of ^18^F-FDG and the detected signal on the PET images [21]. ^18^F-FDG, which has initially arisen in the field of oncology, is nowadays used routinely for inflammatory and infectious diseases [22]. 

In the setting of cardiac imaging, an important parameter is the metabolic fuel of the myocardium on the day of the exam. Indeed, the myocardial metabolism consists mainly of a balance between glucose and free fatty acids [23]. Depending on several physiological and pathological factors, the cardiac metabolism can predominantly switch to glucose, a situation characterized by a diffuse myocardial ^18^F-FDG uptake. A diffuse myocardial ^18^F-FDG uptake can mask a pathologic focal ^18^F-FDG uptake, for example located on a cardiac valve, thereby inducing false negatives. To avoid this, several tools have been developed, considering prior fasting conditions, diet, and blood insulin levels [24]. Carbohydrate consumption prior to the exam leads to increased insulinemia, which activates the expression of GLUT transporters at the surface of cardiomyocytes, favoring a predominantly glucose heart metabolism. Conversely, a high fatty diet will inhibit glucose metabolism and switch the cardiomyocyte metabolism towards free fatty acids consumption. Therefore, the European guidelines recommend specific cardiac preparation before cardiac ^18^F-FDG-PET, which will be detailed in a specific part of this review. 

### 2.2. WBC Scintigraphy

Radiolabeling leukocytes allows tracking their accumulation in infectious sites, making WBC scintigraphy a widely used tool for the detection of infection. Two main radiotracers are available to label WBC: ^111^Indium-oxine (^111^In), which is the first historical tracer in this indication, and ^99m^Technetium-hexamethylpropyleneamine oxime (^99m^Tc-HMPAO) [25,26]. However, ^99m^Tc-HMPAO is currently preferred, owing to its higher image quality (higher signal/noise ratio and spatial resolution), and lower radiation exposure compared to ^111^In [24]. Both ^99m^Tc-HMPAO and ^111^In are lipophilic, a property which enables them to penetrate through the WBC membrane, before attaching to cytoplasmic components. To selectively label WBC, a sample of ca. 50 mL of blood is collected; WBC (either only granulocytes alone or all leukocytes) are separated from other blood cells; cells are incubated with the radiolabeled tracer (^111^In-oxine or ^99m^Tc-HMPAO) and then reinjected in a vein of the patient. This whole procedure should be performed in sterile conditions. In addition, it is recommended to avoid radiolabeling WBC of different patients the same day on the same location, to prevent transfusion accident [25,26]. 

## 3. Diagnostic Performances

### 3.1. F-FDG PET/CT

The performances of ^18^F-FDG-PET/CT highly depend on the type of IE [27]. Therefore, we will distinguish in the following section the different clinical situations.

#### 3.1.1. Native Valve Endocarditis

The literature that specifically evaluated the role of ^18^F-FDG-PET/CT in NVE is limited. A recent meta-analysis identified seven studies addressing this issue, amongst which only two focused solely on patients with a suspicion of NVE, the other consisting of mixed populations of suspected NVE and PVE [28]. All studies were performed following a specific cardiac preparation protocol and all images were acquired 60 min after tracer injection, except for one performed 45 min after injection. While the overall Se of NVE detection was below 50% for most studies (ranging from 0% to 67.7%), the specificity (Sp) was excellent, reaching 100% in five studies. The diagnostic accuracy ranged from 61.8 to 85.2%. The authors calculated pooled Se and Sp of respectively 36.3% and 99.1%. The pooled positive and negative likelihood ratios (PLR and NLR) were respectively of 8.3 and 0.6, with a diagnostic odds ratio (OR) of 15.3. Similar results were reported in a meta-analysis by Wang et al., with pooled Se of 31.0% and Sp of 98% [29]. 

In practice, echocardiography outperforms ^18^F-FDG-PET/CT for the detection of intracardiac evidence of IE. In a prospective study carried out with 120 patients with suspected IE, including 34 NVE, TEE showed a 95.0% Se for the detection of NVE versus 47.6% for ^18^F-FDG-PET/CT [27]. Nevertheless, the addition of ^18^F-FDG-PET/CT may be useful in patients with NVE to detect peripheral FDG uptake corresponding to septic emboli that are often missed by conventional imaging and are considered as a minor criterion of IE in the modified Duke-Li criteria [12]. Consequently, adding ^18^F-FDG-PET/CT in patients with NVE improves the Se of the modified Duke-Li criteria without affecting its high Sp [30,31,32,33]. The prospective multicenter TEPvENDO study reported that, in addition to reclassifying patients with NVE, ^18^F-FDG-PET/CT resulted into a change in the therapeutic management (antibiotic or surgical strategy) in about one third of patients [32]. 

Several explanations account for the low Se of ^18^F-FDG-PET/CT in NVE. While PVE are often related to inflammatory perivalvular abscesses, NVE frequently consist of small (<10 mm) fibrotic vegetations on the valve, with low inflammatory infiltration [1,31]. The relatively low spatial resolution of PET imaging (~5mm) represents an important limitation for the detection of small vegetations with continuous cardiac movements. The Se of ^18^F-FDG-PET may be improved by respiratory and ECG-gated cardiac PET acquisitions compared to static PET acquisitions [34]. The sensitivity of ^18^F-FDG-PET imaging for cardiac infective foci is further decreased in case of failure to suppress ^18^F-FDG uptake in the myocardium. In a study by Abikhzer et al., the exclusion of patients with inadequate myocardial ^18^F-FDG suppression from the analysis resulted in an increase of the Se of ^18^F-FDG-PET/CT with preserved high Sp [30]. Because of the low Se of ^18^F-FDG-PET and the high Se of TEE, ^18^F-FDG-PET/CT is not recommended as a first-line exam for the diagnosis of NVE [12], but may help in case of inconclusive TEE. 

#### 3.1.2. Prosthetic Valve Endocarditis

The literature on the role of ^18^F-FDG-PET/CT for the diagnosis of PVE is increasing at a rapid pace [29,35,36,37]. A recent meta-analysis including 15 studies with 333 cases of PVE showed respective pooled Se and Sp of 86% and 84%, and respective PLR and NLR of 3.23 and 0.21 with a diagnostic OR of 22.0 [29]. Interestingly, the performances of ^18^F-FDG-PET/CT are comparable for mechanical and biological prosthetic valves [38,39,40]. A study in 72 patients with suspected PVE showed that using ^18^F-FDG cardiac uptake as a major criterion improved the Se of the Duke-Li criteria from 70% to 97%, mainly by decreasing the number of PVE initially classified as *possible* IE, and correctly reclassifying them into *definite* PVE [38]. More recently, a prospective study performed in patients with either *definite* or *rejected* PVE showed that adding ^18^F-FDG-PET/CT cardiac findings as a major criterion increased the Se from 57.1% to 83.5%, but at the expense of a reduced Sp from 95.8% to 70.8% [41]. The diagnostic performances of ^18^F-FDG-PET/CT are highly dependent on the preparation of the patient prior to the exam. A recent meta-analysis reported higher performances of ^18^F-FDG-PET/CT when a prolonged cardiac preparation protocol was systematically organized prior to imaging (Se and Sp of 81.3% and 79.0%, respectively) in comparison to former studies inconsistently advising patients to follow the specific diet before imaging (Se 72.3% and Sp 76.2%) [37]. Similar to NVE, ^18^F-FDG-PET/CT offers to identify extracardiac infectious locations of PVE, which are then classified as a minor criterion of the modified Duke-Li criteria [12]. In addition, the presence of increased ^18^F-FDG uptake in the spleen and bone marrow in patients with high likelihood of IE has been shown to be an indirect sign of IE [41,42]. Noteworthy, ^18^F-FDG-PET/CT may still prove useful to detect PVE in case of slow-growing bacteria and in patients with negative blood cultures [43,44,45].

The use of antibiotics prior to imaging influences the diagnostic performance of ^18^F-FDG-PET imaging in IE. The intensity of systemic and local inflammation decreases in parallel to the duration of antibiotherapy, resulting in false-negative ^18^F-FDG-PET/CT results [46,47,48]. The timing of imaging after prosthetic valve surgery is also important [24,49]. Indeed, the healing of tissues after surgery generates local inflammation, which can lead to false positive findings. In addition, surgical adhesives and glue induce a sustained inflammatory reaction in the surgical site [50], which may persist several years after prosthetic valve implantation [46,51,52,53]. Consequently, the European Guidelines recommend performing ^18^F-FDG-PET/CT after an empirical minimal delay of 1–3 months following surgery [12,52], a delay that can be reduced to <3 weeks in case of non-complicated valve surgery and depending on the risk of infection [24]. 

Given its high sensitivity and specificity, the 2015 European guidelines recommend ^18^F-FDG-PET/CT in patients with suspected PVE and diagnostic uncertainty, i.e., PVE classified as *probable* or as *rejected but with persistent high clinical suspicion* based on the Duke-Li criteria [12]. 

An alternative to ^18^F-FDG-PET/CT in case of diagnostic uncertainty is computed tomography angiography (CTA) [12], which can show vegetations on valve leaflets [54]. However, ^18^F-FDG-PET/CT can detect early inflammatory signs before the apparition of anatomical modifications [46]. Combining ^18^F-FDG-PET with CTA improves the diagnostic performances compared to PET with nonenhanced CT (respective Se, Sp, positive predictive value (PPV) and negative predictive value (NPV) of 91%, 90.6%, 92.8%, and 88.3%, versus 86.4%, 87.5%, 90.2%, and 82.9%). In addition, ^18^F-FDG-PET/CTA significantly reduces the rate of doubtful cases from 20% to 8% [39]. CTA is in fact particularly performant to detect complications of PVE, such as pseudoaneurysms and perivalvular abscesses [46,55]. CTA also improves the visualization of valvular thrombi/vegetation as well as the detection of septic emboli [24]. Therefore, ^18^F-FDG-PET/CTA is interesting to detect complications and coronary arteries involvement prior to surgical treatment [46]. Transcatheter-implanted aortic valve (TAVI) procedure is an increasingly used method of valve replacement, especially in the elderly population [56]. TAVI can be complicated by IE [57], a situation where ^18^F-FDG-PET/CTA could be useful. Indeed, detection of TAVI-related IE by echocardiography is limited due to metal artifacts. A recent study showed that while ^18^F-FDG-PET with nonenhanced CT had a low Se to diagnose TAVI-related IE (58%), adding CTA significantly improved the Se (83.3%), reclassifying patients with *possible* IE to either of the two other groups (*definite* or *rejected* IE) [58].

#### 3.1.3. Cardiac Implanted Electronic Device Infective Endocarditis (CIED-IE)

Several studies have specifically investigated the performances of ^18^F-FDG-PET/CT for the diagnostic of CIED-IE [59,60,61,62,63,64]. Two recent meta-analysis reported respective Se 83% and Sp 89%, and Se 87% and Sp 94% [36,59]. Although ^18^F-FDG/PET/CT consistently increases the diagnostic accuracy of the modified Duke-Li criteria, its overall Se remains low in CIED-IE [48,65]. In fact, a distinction must be made between CIED-IE involving the extracardiac components of the device (pocket, extracardiac portion of the leads) and CIED-IE involving the intracardiac portion of the leads [49]. In case of insufficient metabolic preparation, the myocardial uptake of ^18^F-FDG may mask a lead infection, resulting in false negatives [64]. Comparing the performances of ^18^F-FDG-PET/CT in these two settings, Jeronimo et al. reported a Se 72.2% and Sp 95.6% for the diagnosis of pocket infection vs. Se 38.5% and Sp 98.0% for lead infection, despite adequate myocardial suppression in both groups [66]. This is in line with the results of a meta-analysis, reporting the results of subgroup analysis obtained from studies incorporating both pocket and lead IE and showing respective Se 96% and Sp 97% for pocket IE vs. Se 76% and Sp 83% for lead IE [59]. Additional explanations for the false-negative findings in lead infection include prior antibiotherapy and the small size of lead vegetation [66]. In case of suspicion of lead infection, delayed acquisitions (3 h post injection) could improve the diagnostic accuracy compared to standard imaging (70% vs. 51%, respectively) [67]. Additionally, combining ^18^F-FDG-PET with CTA performs better than nonenhanced CT, reclassifying more patients initially deemed *possible* IE and detecting more patterns of IE than nonenhanced CT [39,68]. Furthermore, ^18^F-FDG-PET/CTA may help distinguishing infectious from inflammatory periprosthetic 18F-FDG uptake [69]. 

#### 3.1.4. Left Ventricular Assistance Device Infective Endocarditis (LVAD-IE)

Left ventricular assistant devices (LVAD) are a circulatory support therapeutic option for end-stage heart failure, often in anticipation of heart transplantation. LVAD usually consist of two main parts, which both can be infected: a pump implanted at the left ventricle apex and a driveline. Device infection can occur in about one out of five patients with LVAD [70], and is associated with high morbi-mortality [71]. Diagnosing LVAD-IE can be challenging, and ^18^F-FDG-PET/CT can be helpful in this setting [72]. In a recent meta-analysis by Ten Hove et al. [73], pooled results of 8 studies including 256 exams found Se, Sp, NLR, and PLR, and diagnostic OR of 95%, 91%, 0.14, 3.54, and 38.43 for the diagnosis of either pocket and/or driveline infection, respectively. Similarly high performances were reported by Tam et al., with respective Se and Sp of 92% and 83%, and an AUC of 0.94 [74]. Focusing on driveline infection, ^18^F-FDG-PET/CT’s corresponding performances were 97%, 99%, 3.93, 0.13, and 92.46, respectively. For pump infection, the corresponding diagnostic performances were 97%, 93%, 0.12, 5.56, and 49.43. ^18^F-FDG-PET/CT stigmas of LVAD IE are also associated with an increased mortality, in particular in case of central infection [75]. Interestingly, using the metabolic volume performs better than SUVmax and visual grading for the diagnosis of LVAD-IE [76]. 

#### 3.1.5. Vascular Graft Infection 

The diagnosis of vascular graft infection (VGI), which includes native vessel and endoprosthetic infections, is challenging. Symptoms are often nonspecific and obtaining direct culture material from the vessel is risky. ^18^F-FDG-PET/CT can help in this setting [77,78], with respective pooled Se and Sp of 90–95% and 59–81%, depending on the diagnostic criteria [79]. Comparing three different diagnostic methods, i.e., ^18^F-FDG uptake intensity, ^18^F-FDG pattern, and SUVmax, a focal ^18^F-FDG uptake pattern is the most accurate method for diagnosis of VGI [79].

### 3.2. WBC Scintigraphy

#### 3.2.1. PVE and NVE

Data about the usefulness of WBC scintigraphy in IE are limited and mostly retrospective and mono-centric. In a landmark study by Erba et al. [80] in 51 patients with suspected IE (16 on native valves, 35 on prosthetic valves), WBC scintigraphy showed a 90% Se and a 100% Sp. No differential analysis based on the type of valve (NVE, PVE) was reported in this study. In a preliminary study, we reported the added value of WBC for PVE with inconclusive TTE [81]. In a subsequent study performed in 39 patients with suspected PVE, WBC scintigraphy showed respective Se, Sp, PPV, NPV, and accuracy of 64%, 100%, 100%, 81%, and 86% [82]. The high Sp of WBC scintigraphy was confirmed in three subsequent studies, with respective Sp of 88%, 100%, and 87% [48,83,84]. In the study by Kooshki et al. where all WBC-SPECT results were confronted to surgical findings, adding the results of WBC-SPECT to the modified Duke-Li score correctly re-classified 25% of patients from *possible* to *definite* PVE [83]. Interestingly, this study showed that the intensity of ^99m^Tc-WBC uptake depends on the type of infection, with high signal in abscesses and low signal in non-abscessed lesions, which could partly explain the relatively low Se of WBC scintigraphy [83]. The performances of WBC scintigraphy can also be decreased by former initiation of antibiotherapy. Consequently, WBC scintigraphy should be performed as early as possible to avoid false negatives [48]. In addition, high intensity uptake is associated with a worse outcome, which could have prognostic value and help defining the best management strategy [81,84]. SPECT-based imaging of IE could also benefit from the development of cardiac-dedicated cameras based on cadmium-zinc-telluride (CZT) detectors. CZT cameras offer higher sensitivity than classical Anger cameras thanks to a higher photon counting sensitivity and to the heart-focused disposition of detectors [85,86]. Compared to planar WBC-SPECT, CZT WBC-SPECT significantly improves the detection of WBC signal in patients with IE, with respective Se of 83% vs. 58%, and Sp of 95% vs. 70% [87]. A meta-analysis pooling the results of studies performed with either planar SPECT [80,81,82] and CZT [87] cameras found respective pooled Se and Sp of 86% and 97%, and an excellent accuracy with an area under the curve of 0.957 [36]. 

#### 3.2.2. CIED-IE, LVAD-IE and VGI

Few studies have specifically investigated the diagnostic value of WBC scintigraphy in CIED-IE and/or in LVAD-IE [48,65,88,89]. The reported diagnostic performances range within Se 60–93.7% and Sp within 81–100%. The additional value of WBC-SPECT/CT is particularly marked in case of CIED-IE deemed as *possible* based on the Duke-Li criteria [48]. Adding the results of WBC-SPECT to the Duke criteria improved the diagnostic accuracy from 83% to 88% [65]. A small study performed in patients with LVAD-IE showed a 100% Se with no false positive results [90]. A more recent study reported performances comparable to those in CIED-IE, with respective Se, Sp, PPV, NPV, and accuracy of 71.4%, 100%, 100%, 33.3%, and 75% [88]. In the setting of suspected VGI, the diagnostic performances of ^99m^Tc-WBC-SPECT range within Se 82–100% and Sp 75–100% [89]. The performances remain in case of late or a low-grade late prosthetic VGI [91] and when SPECT/CT is performed within the first month after surgery [92]. 

## 4. Septic Emboli

Septic emboli are caused by bloodstream spreading of the infection to distant locations, with the most common locations being cerebral and pulmonary [1]. Risk factors associated with septic emboli include left-sided IE, pulmonary endocarditis, bivalvular NVE, intracardiac CIED, vegetation, fungal infection, and *Staphylococcus aureus* infection [1,5,93]. Septic emboli are together with heart failure the most frequent complications of IE, involving one out of four patients at admission [1,2]. After the initiation of antibiotic treatment, the prevalence of septic emboli remains high, reported in 20% of hospitalized IE patients [1]. In fact, septic emboli may remain clinically silent [94]. In addition, conventional imaging often miss distal septic foci [95]. Consequently, the true prevalence of septic emboli could be much higher than 25% [96]. The detection of septic emboli may impact treatment strategy, generally a prolongation of antibiotic treatment, but also the decision for early surgery in about half the cases [1]. Failure to identify septic emboli can thus lead to insufficient treatment and worsened outcomes, either relapse or even death [93]. Since most septic emboli occur within the two first weeks after treatment initiation [97], an early diagnosis is crucial. Nuclear imaging offers the substantial advantage of enabling cardiac and extracardiac evaluation in a single exam. 

### 4.1. F-FDG-PET

Several studies have reported the high diagnostic value of ^18^F-FDG-PET/CT for the early detection of septic emboli from IE [98]. In a population of 25 patients with IE, ^18^F-FDG-PET/CT evidenced septic emboli in 40% of patients, of which 28% were clinically silent, and correctly excluded distant localizations in 56% of cases [96]. Similar results were reported in 2 subsequent studies performed in 72 patients with suspected IE each, both studies showing that ^18^F-FDG-PET revealed clinically silent distant foci in 24% of cases, leading to antibiotherapy initiation or prolongation in all cases [99,100]. Compared with a historical cohort of patients with IE (NVE, PVE, CIED-IE), ^18^F-FDG-PET detected septic emboli with respective Se, Sp, PPV, and NPV of 100%, 80%, 90%, and 100% [95]. Importantly, ^18^F-FDG-PET evidenced significantly more septic emboli than the conventional approach, including other imaging methods (57.4% vs. 18.0%) [95]. Noteworthy, in this study ^18^F-FDG-PET was also the only initially positive imaging finding in 55.5% of cases [95]. This early detection of septic emboli resulted into the prolongation of antibiotherapy in these patients and a two-fold reduction of the incidence of relapses compared with the historical cohort [95]. In a study specifically investigating the therapeutic implications of detecting septic emboli, ^18^F-FDG-PET modified the therapeutic strategy in 35% of patients, including antibiotics prolongation, surgery, and prevention of unnecessary extraction of material [94]. In the TEPvENDO study, while the rate of extracardiac foci was similar in NVE and PVE, the impact of detecting septic emboli on the management was more evident in NVE than in PVE [32]. The diagnostic performances of PET also depend on the organ’s physiological ^18^F-FDG uptake. In 72 patients with definite IE, Özcan et al. showed that in organs with high physiological FDG uptake (including brain and heart), the Se of ^18^F-FDG-PET to diagnose infectious localizations was as low as 13%, compared with 87% in organs with low physiological uptake, resulting in an overall Se 40% and PPV 56% [101]. The low performance of ^18^F-FDG-PET/CT in the detection of brain foci is particularly problematic given the high risk of cerebral complications, which has been reported in up to 25% of IE patients [102]. On standard ^18^F-FDG-PET acquisitions, performed 60 min after tracer injection, the physiological brain ^18^F-FDG uptake is high, which can mask potential cerebral infectious foci. To overcome this, additional PET images centered on the head can be acquired 180 min after ^18^F-FDG injection, at a time where physiological brain glucose consumption is lower, and pathological ^18^F-FDG uptake may be detected with a higher contrast [103]. Performing systematic brain-centered late images has been shown to enable the detection of cerebral IE complications in up to 8.8% [32]. Nevertheless, conventional imaging and especially magnetic resonance imaging remains essential in this indication [104]. A classical complication of IE is mycotic aneurysm, i.e., a septic graft that can develop on a peripheral vessel. Mycotic aneurysms may develop along the whole arterial system, including in the lower limbs, and may evolve towards fatal rupture if not identified and treated at an early stage [105]. Therefore, including the lower limbs in the field of PET acquisitions is useful to detect asymptomatic mycotic aneurysms and initiate early treatment [106]. Similar to NVE and PVE, ^18^F-FDG-PET/CT is also highly performant to evidence septic emboli originating from cardiovascular devices [98]. Several studies performed in patients with CIED have demonstrated that ^18^F-FDG-PET/CT could reveal unknown extracardiac infectious foci in about one out of five patients [39,64,107,108,109]. 

### 4.2. WBC Scintigraphy

WBC scintigraphy offers the advantage of allowing in a single scan to identify both cardiac and extracardiac infection [35,89]. In a study including 51 patients with IE, WBC scintigraphy detected extracardiac uptake in 24 patients, correctly identifying septic embolism in 21 of them [80]. In 31 patients with CIED-IE, the same team showed that WBC scintigraphy correctly identified 6 cases of metastatic infection [110]. This is in agreement with the more recent studies showing a septic emboli detection rate of 47.5% in IE [111] and 34% in CIED-IE [65], respectively.

## 5. Portal of Entry

Identifying and treating a portal of entry of IE is crucial, since these may lead to the recurrence of sepsis and IE [112]. In addition, the infectious portal of entry may in some cases be caused by a chronic disease requiring a dedicated treatment in addition to IE. The most common infectious portal of entries of IE are cutaneous (notably in patients using intravenous drugs, classically leading to right-sided IE) and digestive (in particular, colic cancer and oral/dental infection) [113,114,115]. ^18^F-FDG-PET imaging offers high Se for the detection of cancer [21] and infectious foci [22] and appears therefore well-suited for the identification of the portal of entry in patients with IE [98,104,113]. In a study specifically assessing the incidence of cancer during IE, a cancer was found with ^18^F-FDG-PET/CT in 7.5% of patients [116]. The TEPvENDO study reported an overall 23.6% detection rate of portal of entry, either oncologic or infectious causes [32]. In a study in 114 IE patients evaluating the ability of ^18^F-FDG-PET to detect extra-cardiac foci, 74 new extra-cardiac findings were reported on PET that were not previously discovered by other modalities (mainly cancer and infection foci), leading to a change in treatment in 10% of patients [117]. 

## 6. Prognosis

In patients with PVE, positive ^18^F-FDG valvular uptake is associated with worse cardiovascular outcomes, i.e., of death, IE recurrence, acute heart failure, nonscheduled cardiovascular hospitalization, and new embolic event [118]. Interestingly, this association was stronger in case of moderate to intense uptake compared with negative or low uptake. In patients with NVE, a moderate to intense valvular ^18^F-FDG uptake was significantly associated with more frequent new embolic events [118]. The prognostic value of ^18^F-FDG-PET/CT in CIED-IE is less documented. A recent study found that patients with confirmed lead CIED-IE but *without* ^18^F-FDG uptake around the pocket nor clinical signs of pocket infection (“cold close pocket” infection) experienced worse outcome following lead extraction [119]. If confirmed, these results might indicate a prognostic value for ^18^F-FDG-PET/CT prior to lead extraction. Regarding WBC-SPECT, a study evaluating its prognostic role in patients with suspected CIED-IE found that all-cause mortality rates did not significantly differ between patients with positive WBC-SPECT findings and those with negative results [84]. However, positive WBC-SPECT results were associated with higher in-hospital mortality, complication rate, and frequency of hardware removal [84]. Noteworthy, the increase in the use of nuclear imaging (both ^18^F-FDG-PET and WBC scintigraphy) in patients with IE is associated with a reduced time to cardiac surgery [120]. 

## 7. Comparison of ^18^F-FDG-PET/CT and WBC-SPECT Imaging in IE

### 7.1. Diagnosis

The abovementioned studies showed an overall high sensitivity of ^18^F-FDG-PET/CT for the diagnosis of IE, and a high specificity of WBC-SPECT scintigraphy. Several studies have compared head-to-head the performance of both techniques. In a retrospective study of 39 patients with suspected PVE undergoing both ^18^F-FDG-PET/CT and WBC-SPECT imaging, the respective Se, Sp, PPV, NPV, and accuracy were 93%, 71%, 68%, 94%, and 80% for ^18^F-FDG PET, and 64%, 100%, 100%, 81%, and 86% for WBC-SPECT scintigraphy [82]. In 48 patients with suspected CIED-IE, the respective Se, Sp, PPV and NPV were 80%, 91%, 80%, and 91% for ^18^F-FDG-PET/CT, and 60%, 100%, 100%, and 85% for WBC scintigraphy [48]. Similarly, in 24 patients with suspected LVAD-IE, the respective Se, Sp, PPV, NPV, and accuracy were 95.2%, 66.7%, 95.2%, 66.7%, and 91.6% for ^18^F-FDG-PET, and 71.4%, 100%, 100%, 33.3%, and 75% for WBC-SPECT scintigraphy [88]. However, in patients with suspected VGI, the diagnostic performances of WBC-SPECT scintigraphy are higher than ^18^F-FDG-PET [79]. One study comparing head-to-head both techniques showed overall better performance for WBC-SPECT scintigraphy, with respective ROC AUC of 0.902 for WBC scintigraphy, and 0.759 for ^18^F-FDG-PET [121]. The corresponding diagnostic performances were respectively Se 89.5%, Sp 90.9%, PPV 70.8%, NPV 97.2% and accuracy 90.6% for WBC-SPECT scintigraphy, and Se 85%, Sp 68.4%, NPV 41.5%, PPV 94.5%, and accuracy 71.9% for ^18^F-FDG-PET. Consequently, a two-step approach may be recommended in patients with suspected IE/CIED with ^18^F-FDG-PET imaging as first line imaging technique thanks to its high sensitivity and relative availability, and WBC-SPECT as the second line imaging technique in patients requiring discriminating infective from inflammatory causes of the ^18^F-FDG uptake taking advantage of its high specificity for infection. 

### 7.2. Septic Emboli

Lauridsen et al. compared the respective performances of ^18^FDG-PET/CT and WBC-SPECT/CT in the detection of clinically relevant extra-cardiac manifestations of IE [122]. ^18^F-FDG-PET/CT proved superior to WBC-SPECT scintigraphy, correctly identifying 91 foci in 32 positive scans for ^18^F-FDG-PET, and 37 foci in 24 positive scans for WBC-SPECT, resulting in a mean sum of identified foci of 2.57 vs. 1.06, respectively. The clinical impact of the detection of extra-cardiac localizations was also significantly higher for ^18^F-FDG-PET compared to WBC-SPECT. 

## 8. Practical Approach 

### 8.1. F-FDG-PET/CT

#### 8.1.1. Patient Preparation

In addition to prolonged fasting prior to image acquisition, which is the standard for all ^18^F-FDG-PET imaging, it is important to reduce the myocardial physiological glucose metabolism. The protocol recommended by guidelines issued by the European Association of Nuclear Medicine and the European Association of Cardiovascular Imaging consists of a high-fat/low carbohydrates diet (HF/LCD) for 12–24 h combined with a prolonged fasting period of 12–18 h [24]. In addition, the intravenous administration of 50 IU/kg of heparin 15 min prior to ^18^F-FDG injection may help decrease myocardial uptake of ^18^F-FDG, but the evidence is not as strong [123,124,125]. A recent study described that the injection of an intravenous lipid emulsion before FDG injection in combination with the HF/LCD resulted into a higher rate of FDG uptake in the myocardium compared to HF/LCD alone [126].

#### 8.1.2. Acquisition

In case of suspicion of PVE or CIED-IE, the analysis in the first three months after surgery should be cautious, in particular if surgical adhesives have been used [24,46]. The recommended ^18^F-FDG activity for IE imaging is 2.5–5.0 MBq/Kg and should be adapted to the intrinsic sensitivity of the PET system [24]. Images are usually acquired 60 min post-injection [24]. To perform a complete analysis (including brain and lower limbs), whole-body acquisitions from head to toes are useful, instead of the head to mid-thigh acquisitions classically performed for oncological indications [32,106]. In addition, performing additional brain-centered late acquisitions 3 h after ^18^F-FDG injection can improve the detection of brain foci [32]. If local expertise and technical requirements are available, performing CTA instead of non-enhanced CT can be useful, especially in patients with aortic grafts, or congenital heart diseases and complex anatomy [24,68]. If PET/CTA is performed, ECG-gating is recommended with at least a 64-detector row CT [24]. An arterial phase imaging is recommended for left-sided IE, and a venous phase imaging for suspected CIED-IE to evaluate soft tissue changes, lead vegetation, and venous thrombosis of the vascular accesses [24]. 

#### 8.1.3. Image Analysis

##### Cardiac Analysis

**Figure 1:**
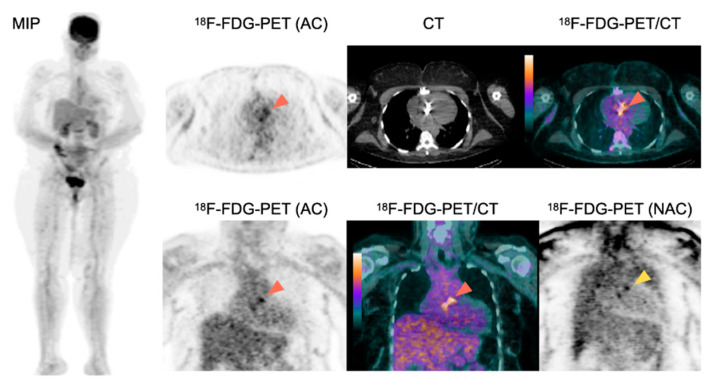
Role of ^18^F-FDG-PET/CT for the diagnosis of PVE.

Typical features of IE consist of focal and heterogenous valvular/perivalvular uptake. Abscesses can develop in area in contact to prosthesis, but the most typical location is the aorto-mitral trigon. The higher ^18^F-FDG uptake, the higher the probability of IE, except in regions where surgical glue has been used [24,127]. In case of suspected PVE or CIED-IE, the presence of material can cause artifacts due to over-correction of signal attenuation. Therefore, in this situation it is important to analyze both attenuation-corrected (AC) and non-attenuation corrected (NAC) acquisitions. Periprosthetic ^18^F-FDG uptake on AC images persisting on NAC images is suggestive of PVE (Figure 1). 

A 63-year-old female patient with history of aortic valve replacement presented with a fever of unknown origin. Two blood cultures taken 12 h apart were positive for *Staphylococcus aureus*. ^18^F-FDG-PET/CT showed an intense ^18^F-FDG uptake around the aortic prosthetic valve on AC images (orange arrowheads), persisting on NAC images (yellow arrowhead) suggestive of PVE. The presence of two major criteria for IE classified the patient as having *definite IE* based on the modified Duke-Li criteria. ^18^F-FDG-PET showed no sign of septic emboli. Consequently, the patient underwent a prosthetic valve replacement surgery and a prolonged antibiotherapy.

**Figure 2:**
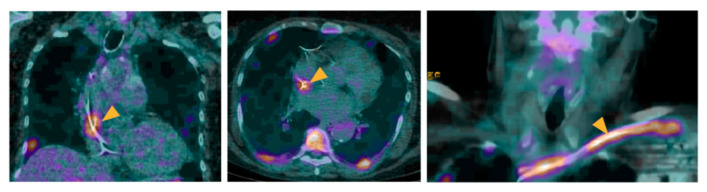
Role of ^18^F-FDG-PET/CT in a patient with a suspicion of IE on CIED.

Conversely, the homogeneous perivalvular ^18^F-FDG signal, which is only present on AC images but not on NAC images, is less suggestive of PVE [24]. In case of a suspicion of CIED or LVAD infection, focal/linear ^18^F-FDG uptake around the material on AC acquisitions, particularly when facing a lead/the pump, and which persists on NAC images, is suggestive of IE [24] (Figure 2). In case of a suspicion of VGI infection, high ^18^F-FDG uptake, especially when developing on an underlying vascular abnormality (aneurysm, calcification) is suggestive of infectious graft [24,78]. The use of quantitative and semiquantitative methods based on SUV have been proposed [46,63,72,128,129,130], but are currently not routinely used. 

A 55-year-old male patient with a history of pacemaker implantation presented with a fever of unknown origin. No clinical sign of pocket infection was observed. Blood culture tested positive for *Streptococcus viridans*. Echocardiography evidenced no sign of CIED-IE. ^18^F-FDG-PET/CT showed an intense ^18^F-FDG uptake located at the level of the extra-cardiac portion of the lead (arrowheads), suggestive of a lead infection. The presence of two major criteria classified the patient as with *definite IE*. The patient further underwent an extraction of the device, which culture confirmed the diagnosis of CIED-IE, as well as prolonged antibiotherapy. 

##### Extra-Cardiac Analysis

**Figure 3:**
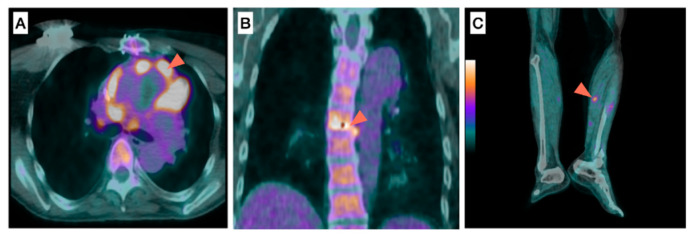
Role of ^18^F-FDG-PET/CT for the detection of septic emboli in a patient with infective endocarditis. (**A**) prosthetic valve, (**B**) spine, (**C**) anterior tibial artery.

The detection of extracardiac focal ^18^F-FDG uptake, suggestive of septic emboli, reinforces the probability of IE [12]. Typical locations for septic emboli are the spleen, the liver, the lungs, the spine, and the kidneys [24] (Figure 3). Extracardiac focal FDG uptake may also be caused by a portal of entry (infectious or malignant). Typical locations for portals of entry are the colon, skin, sinuses, and dental abscesses [24]. Spleen and bone marrow ^18^F-FDG uptake in patients with high likelihood of IE can also reinforce the diagnostic suspicion [41,42].

A 60-year-old male patient with a history of aortic valve prosthetic replacement presented with fever and two blood cultures positive for *Staphylococcus aureus*. ^18^F-FDG-PET/CT showed an intense ^18^F-FDG uptake around the aortic prosthetic valve (Figure 3A, arrowhead) suggestive of PVE, associated to extracardiac ^18^F-FDG uptake located between two vertebras (Figure 3B, arrowhead) in favor of spondylodiscitis as well as along the left anterior tibial artery (Figure 3C, arrowhead) suggestive of mycotic aneurysm. The patient underwent prosthetic valve replacement and antibiotherapy, which duration was prolonged given the presence of septic emboli.

### 8.2. WBC-SPECT 

#### 8.2.1. Patient Preparation

No specific preparation is recommended prior to WBC-SPECT scintigraphy. WBC-SPECT scintigraphy should be performed as soon as possible after initiation of antibiotics to prevent decrease in the intensity of the signal due to treatment [123]. 

#### 8.2.2. Acquisition

After injecting radiolabeled WBC, two series of scintigraphy acquisitions are performed: an early acquisition 4–6 h post-injection, followed by late images, usually acquired 24 h post-injection. [123]. The early signal may be related to both inflammation and infection, whereas the persistence of a signal on late acquisitions is more specific for infection. In addition, background blood signal has decreased on late acquisitions, thereby improving the signal/noise ratio and the contrast of images. Acquisitions usually consist of whole-body planar images and SPECT-CT acquisitions focused on the thorax or on the regions with high signal next to the implanted material. SPECT-CT acquisitions provide higher sensitivity than planar images and allows for the precise location of the signal on fused SPECT-CT images. 

#### 8.2.3. Image Analysis

##### Cardiac Analysis

**Figure 4:**
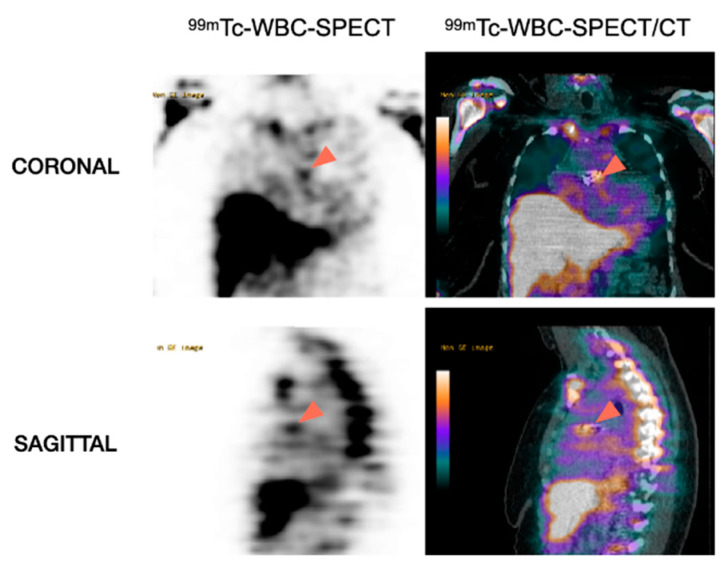
Role of WBC-SPECT/CT for the detection of prosthetic valve endocarditis. Note the accumulation of ^99m^Tc-labeled WBC in the region corresponding to the aortic prosthetic valve (arrowheads) in favor of PVE.

Typical features of IE consist of focal/linear uptake of the valvular/perivalvular area or around the device (Figure 4), visible on the early acquisitions and increasing on the late images [123]. Similar to ^18^F-FDG-PET/CT, both AC and NAC should be analyzed carefully to exclude the artefactual signal related to over-correction of attenuation in regions with implanted material. 

**Figure 5:**
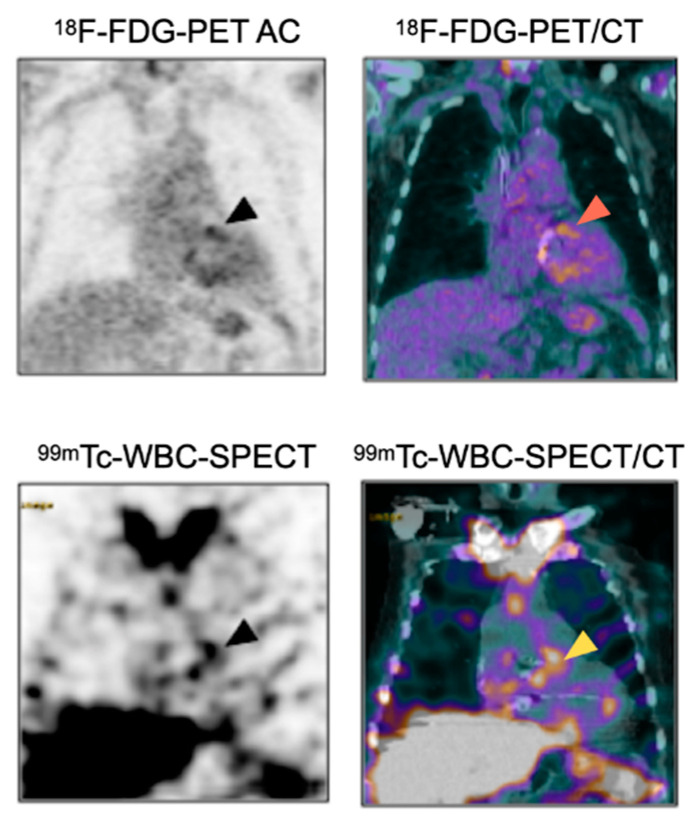
Complementary diagnostic value of ^18^F-FDG-PET/CT and ^99m^Tc-WBC-SPECT/CT for the diagnosis of PVE. Patient with suspicion of aortic PVE one month after surgery. ^18^F-FDG-PET/CT showed intense uptake around the aortic prosthetic valve (arrowheads), which could in this context be related to PVE or to post-surgery inflammatory reaction. ^99m^Tc-WBC accumulation in the same area as the ^18^F-FDG-PET/CT helped to confirm the diagnosis of prosthetic valve infection.

Figure 5 shows a patient with aortic prosthetic valve in whom both ^18^F-FDG-PET/CT and ^99m^Tc-WBC-SPECT/CT have been performed.

###### Extra-Cardiac Analysis

Images suggestive of septic emboli can appear either as areas of increased uptake, or as cold spots, notably in the spleen and spine [123]. 

## 9. Diagnostic Imaging Algorithm

### 9.1. For NVE and PVE

**Figure 6:**
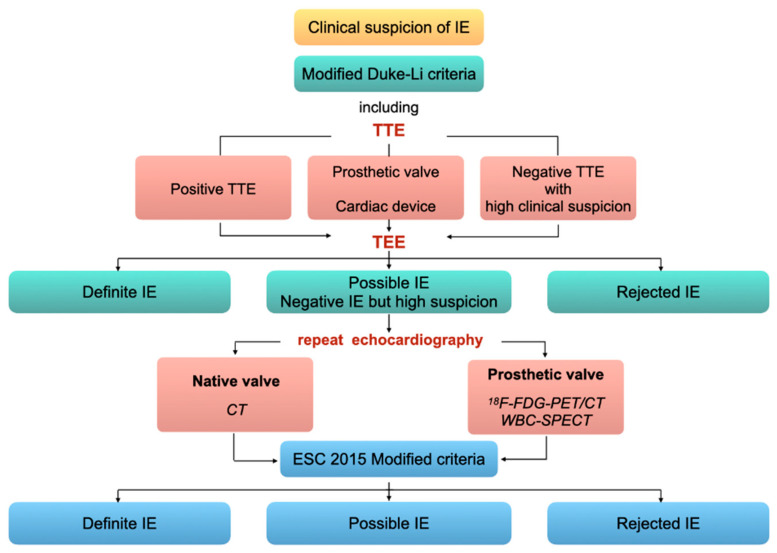
Imaging diagnostic algorithm for valve IE. Adapted from the 2015 ESC guidelines [12].

According to the 2015 European Society of Cardiology guidelines [12], TTE is the first-line exam in case of clinical suspicion of IE. If TTE is in favor of IE, or if it is non-diagnostic, or in case of prosthetic valve/intracardiac device, TEE is recommended (class I, level B). TEE is also recommended in case of negative TTE with high clinical suspicion (class I, level C). Based on clinical, microbiological, and echocardiographic findings, the probability of IE is graded according to the modified Duke-Li criteria as either *definite*, *probable*, or *rejected*. In case of *possible* IE or *rejected* IE but with high suspicion, echocardiography must be repeated, and in patients with prosthetic valve, ^18^F-FDG-PET/CT or WBC-SPECT can be performed. Positive findings on ^18^F-FDG-PET/CT or WBC-SPECT account as major criteria of the European Society of Cardiology 2015 modified diagnostic criteria. Accordingly, the probability of IE is again graded into *positive*, *possible*, or *rejected* (Figure 6). 

### 9.2. For CIED-IE

**Figure 7:**
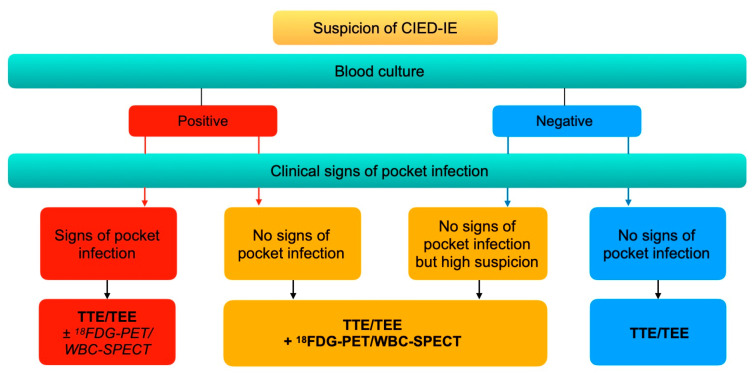
Imaging diagnostic algorithm for CIED-IE. Adapted from the European Heart Rhythm Association international consensus [19].

The European Heart Rhythm Association issued in 2020 an international consensus on the management of CIED-IE [19]. In case of clinical signs of pocket infection, echocardiography (TTE and TEE) is recommended, regardless of the positivity of blood cultures. ^18^F-FDG-PET or WBC-SPECT/CT can optionally be performed. If there are no clinical signs of pocket infection, nuclear medicine investigations (^18^F-FDG-PET/CT or WBC-SPECT/CT) are recommended in case of positive blood culture or of high suspicion of CIED-IE. Additionally, ^18^F-FDG-PET/CT and WBC-SPECT/CT are recommended to assess the presence of septic emboli (Figure 7). 

## 10. Potential Impact of Nuclear Medicine Tools on Treatment Strategy

There are currently no studies specifically evaluating the impact of the results of nuclear imaging on the choice of treatment strategy, which has been identified as an area for study in future research [131]. Yet, ^18^F-FDG-PET/CT and WBC-SPECT/CT could prove useful at several stages of IE management. Indeed, morphological changes induced by IE are predated by the functional changes, which can be evidenced by nuclear medicine imaging. Given the fact that a delayed initiation of therapy is associated with an increased risk of mortality or of septic emboli [132], the early diagnosis by scintigraphy could improve the outcome of IE patients. Nuclear medicine imaging could also help tailor the choice of treatment modality. Outpatient parenteral antibiotic therapy is an option in stable patients with no evidence of complication [12,133]. Consequently, ^18^F-FDG-PET/CT can help exclude extracardiac localizations of IE before opting for outpatient therapy. Conversely, if septic emboli are evidenced on ^18^F-FDG-PET/CT, a more aggressive inpatient treatment is to be preferred, i.e., surgery and/or a prolonged duration of antibiotherapy [12]. In patients in whom antibiotic therapy is decided, ^18^F-FDG-PET/CT could prove useful to assess the disease activity and consequently to monitor the response to treatment [47].

## 11. Conclusions

Nuclear medicine imaging is increasingly used in patients suspected of IE. The 2015 European guidelines have included ^18^F-FDG-PET/CT and WBC scintigraphy findings as major criteria for the diagnosis of IE, in particular in patients with prosthetic valve and inconclusive echocardiography. These techniques also allow a single-shot investigation of septic emboli, and ^18^F-FDG-PET/CT can also detect the potential portal of entry. Given the high sensitivity of ^18^F-FDG-PET/CT and the high specificity of WBC scintigraphy, combining both methods is a promising approach for the diagnosis of IE.

## Figures and Tables

**Table 1:** Modified Duke-Li criteria for the diagnosis of valve infective endocarditis.

Major Criteria	1. Microbiological Criteria
	a. Microorganisms typical of IE evidenced from two separate blood cultures
	- Viridans streptococci, Streptococcus gallolyticus (Streptococcus bovis), HACEK group, Staphylococcus aureus OR - Community-acquired enterococci, in the absence of a primary focus OR
	b. Microorganisms consistent with IE evidenced from persistently positive blood cultures:
	- ≥2 positive blood cultures of blood samples collected >12 h apart OR - 3 or a majority of ≥4 separate positive blood cultures (first and last collected > 1 h apart) OR - Single positive blood culture for Coxiella burnetii or phase I IgG antibody titre >1:800
	2. Imaging Criteria
	a. Echocardiogram positive for IE showing one/several of the following typical findings
	- Vegetation - Abscess, pseudoaneurysm, intracardiac fistula - Valvular perforation or aneurysm - New partial dehiscence of prosthetic valve
	b. *Nuclear medicine imaging positive for IE, i.e., abnormal uptake around the site of prosthetic valve implantation*
	- *On ^18^F-FDG PET/CT if the prosthesis was implanted >3 months* OR - *On radiolabeled WBC-SPECT/CT*
	c. *Cardiac CT*
	- *Paravalvular lesions*
Minor Criteria	1. Predisposing condition such as heart condition, or intravenous drug use
	2. Fever defined as temperature >38 °C
	3. Vascular phenomena *including those detected only by imaging,* major arterial emboli, septic pulmonary infarcts, mycotic aneurysm, intracranial hemorrhage, conjunctival hemorrhages, and Janeway’s lesions
	4. Immunological phenomena: glomerulonephritis, Osler’s nodes, Roth’s spots, and rheumatoid factor
	5. Microbiological evidence: positive blood culture, but does not meet a major criterion as noted above, or serological evidence of active infection with organism consistent with IE

Legend. ^18^F-FDG PET: 18Fluor fluorodeoxyglucose positron emission tomography; CT: computed tomography; HACEK: Haemophilus, Aggregatibacter, Cardiobacterium, Eikenella, Kingella; IE: infective endocarditis; SPECT: single photon emission computed tomography; WBC: white blood cell. *Text in italic font indicates the modifications to the Duke-Li criteria implemented in the 2015 European Society of Cardiology guidelines.* Adapted from Habib et al. [12].

**Table 2:** Definition of infective endocarditis according to the modified Duke criteria. Adapted from Habib et al. [12].

Definite IE	Histopathological Criteria
	Demonstration of a microorganism from a culture, a cardiac vegetation, an embolized vegetation, or an intracardiac abscess, OR Demonstration of an active endocarditis from a vegetation or an intracardiac abscess
	Clinical Criteria
	2 major criteria, OR 1 major criterion AND 3 minor criteria, OR 5 minor criteria
Possible IE	1 major criterion AND 1 minor criterion, OR 3 minor criteria
Rejected IE	Firm alternate diagnosis, OR Resolution of symptoms within ≤4 days of antibiotherapy, OR No pathological evidence of IE (surgery or autopsy) after ≤4 days of antibiotherapy, OR No criteria for *possible IE* as defined above

**Table 3:** Comparison between ^18^F-FDG-PET/CT and WBC-SPECT/CT.

	Advantages	Drawbacks
^18^F-FDG-PET/CT	High sensitivity for PVE and device-related IE (CIED pocket and extracardiac lead)	Moderate sensitivity for NVE and intracardiac lead CIED-IE
	Good spatial resolution (4–5 mm)	Moderate specificity for infection
	Short protocol (preparation and acquisition <2 h)	Requires a specific diet to suppress the physiological cardiac uptake of ^18^F-FDG
	Whole-body imaging in 15–20 min. allowing for the detection of device infection and septic emboli	Post-surgery inflammation in case of PVE (cautious interpretation 1–3 months after surgery)
	Identification of possible portal of entry	Limited sensitivity in organs with high FDG uptake, especially the brain
	Identification of alternate diagnosis for infectious or inflammatory syndrome than IE	Possible false-negative results in small vegetations and/or after prolonged antibiotherapy
		Radiation exposure
WBC-SPECT/CT	High specificity	Moderate sensitivity, especially for CIED-IE
	No need for specific diet nor interaction with sugar levels for imaging	Long and complex procedure requiring blood handling
	Relatively low spatial resolution (8–10 mm)	Possible false-negative results in small vegetations and/or prolonged antibiotherapy
	Lower imaqe quality (late imaging time point and SPECT acquistions)	Radiation exposure
	Potential detection of septic emboli, but lower performance than ^18^F-FDG-PET/CT	

Legend. ^18^F-FDG PET: 18Fluor fluorodeoxyglucose positron emission tomography; CIED: cardiac implantable electronic device; CT: computed tomography; IE: infective endocarditis; NVE: native valve endocarditis; PVE: prosthetic valve endocarditis; SPECT: single photon emission computed tomography; WBC: white blood cell.

**Table 4:** Novel 2019 International Criteria for the diagnosis of CIED-IE.

Major Criteria	1. Microbiological Criteria
	a. Microorganisms typical of CIED-IE and/or IE (Coagulase-negative staphylococci, Staphylococcus aureus)
	b. Microorganisms typical of IE evidenced from two separate blood cultures
	- Viridans streptococci, Streptococcus gallolyticus (Streptococcus bovis), HACEK group, Staphylococcus aureus OR - Community-acquired enterococci, in the absence of a primary focus OR
	c. Microorganisms consistent with IE evidenced from persistently positive blood cultures:
	- ≥2 positive blood cultures of blood samples collected >12 h apart OR - 3 or a majority of ≥4 separate positive blood cultures (first and last collected >1 h apart) OR - Single positive blood culture for Coxiella burnetii or phase I IgG antibody titre >1:800
	2. Imaging Criteria
	a. Echocardiogram positive for CIED-IE:
	clinical pocket/generator infectionlead-vegetation
	b. *Nuclear medicine imaging positive for CIED-IE, i.e., abnormal uptake around pocket/generator site or along leads*
	- *On ^18^F-FDG PET/CT (caution in case of recent implants)* OR - *On radiolabeled WBC-SPECT/CT*
Minor criteria	1. Predisposing condition such as heart condition or intravenous drug use
	2. Fever defined as temperature >38 °C
	3. Vascular phenomena *including those detected only by imaging,* major arterial emboli, septic pulmonary infarcts, mycotic aneurysm, intracranial hemorrhage, conjunctival hemorrhages, and Janeway’s lesions
	4. Microbiological evidence: positive blood culture but does not meet a major criterion as noted above or serological evidence of active infection with organism consistent with CIED-IE

Legend. ^18^F-FDG PET: ^18^Fluor fluorodeoxyglucose positron emission tomography; CT: computed tomography; HACEK: Haemophilus, Aggregatibacter, Cardiobacterium, Eikenella, Kingella; CIED: cardiac implantable electronic device; IE: infective endocarditis; SPECT: single photon emission computed tomography; WBC: white blood cell. *Text in italic font indicates the modifications to the Duke-Li criteria implemented in the 2015 European Society of Cardiology guidelines*. Adapted from Blomström-Lundqvist [19].

**Table 5:** Main advantages/limitations of nuclear/morphological techniques for the diagnosis of IE.

	Echocardiography	CCTA	Cardiac MRI	^18^F-FDG-PET/CT	WBC-SPECT/CT
Diagnostic Performances for IE Diagnosis	- High spatial and temporal resolution - High diagnostic performances in NVE, lower in PVE	- High spatial and temporal resolution - Good performances for the detection of perivalvular lesions in PVE	- Conflicting data about performances in NVE - Limited data about performances in mechanical PVE	- High sensitivity in PVE - Low sensitivity in NVE	- High specificity in PVE and NVE - Low sensitivity in NVE
Evaluation of Cardiac Complications	- Allows precise evaluation of valvular dysfunction and lesions due to IE	- Allows evaluation of perivalvular lesions (abscess-pseudoaneurysm)	- Allows evaluation of myocardial and valvular function	- Limited evaluation of perivalvular extension	- Limited evaluation of perivalvular extension
Cardiac Presurgical Assessment	- Assessment of cardiac function and evaluation of aortic root	- Allows to evaluate aortic root and coronary arteries	- Assessment of cardiac function and aortic root	-	-
Extracardiac Assessment	- No extracardiac workup	- Detection of peripheral embols if combined with wholebody CTA	- No extracardiac workup	- Detection of septic embols, septic aneurysms and protal of entry with high sensitivity	- Detection of septic embols
Contra-Indications	- No contraindication for TTE - Esophageal pathology for TEE	- Pregnancy, allergy to iodinated contrast media, severe renal insufficiency	- Pregnancy, close monitoring in presence of ICD or PM, CI for some old metallic prosthesis, claustrophobia, severe renal insufficiency	- Pregnancy	- Pregnancy
Availability	- Widely and easily available	- Widely available	- Moderate availability	- Moderate availability	- Limited availability
Limitations and drawbacks	- Operator dependent analysis - Metallic artifacts in PVE	- Metallic artifacts in PVE, CIED - Difficulty to discriminate vegetation from thrombus and hematoma from abscess based only on morphological imaging	- Metallic artifacts in PVE - Cardiac and respiratory artifacts	- Lack of specificity - Need for prolonged fasting and dedicated cardiac preparation	- Complex handling of blood products

## Data Availability

Data sharing not applicable.

## Abbreviations

^18^F-FDG	^18^Fluor radiolabeled fluorodeoxyglucose
^111^In	^111^Indium-oxine
^99m^Tc-HMPAO	^99m^Technetium-hexamethylpropyleneamine oxime
AC	attenuation corrected
CIED	cardiac implantable electronic device
CTA	computed tomography angiography
CZT	cadmium-zinc-telluride
HF/LCD	high fat/low carbohydrates diet
IE	infective endocarditis
LVAD	left ventricular assistance device
NAC	non-attenuation corrected
NLR	negative likelihood ratio
NPV	negative predictive value
NVE	native valve endocarditis
OR	odds ratio
PET	positron emission tomography combined with computed tomography
PLR	positive likelihood ratio
PPV	positive predictive value
PVE	prosthetic valve endocarditis
Se	sensitivity
Sp	specificity
SPECT	single photon emission computed tomography
TEE	transesophageal echocardiography
TTE	transthoracic echocardiography
VGI	vascular graft infection
WBC	white blood cell
^18^F-FDG-PET	^18^F-fluorodeoxyglucose positron emission tomography
^99m^Tc-WBC	^99m^Technetium radiolabeled white blood cells
AC	attenuation correction
CIED	cardiac implanted electronic device
CT	computed tomography; IE: infective endocarditis
MIP	maximal intensity projection
NAC	non-attenuation correction
PVE	prosthetic valve endocarditis
SPECT	single photon emission computed tomography
TEE	transesophageal echocardiography
TTE	transthoracic echocardiography.
